# Closing the global surgical divide: histotripsy, a pathway to universal access?

**DOI:** 10.1097/MS9.0000000000005226

**Published:** 2026-06-08

**Authors:** Obi Ekwenna, Naoru Koizumi, Aidin Abedi, Tarak Davuluri

**Affiliations:** a Department of Urology and Transplantation, University of Toledo College of Medicine and Life Sciences, Toledo, OH, USA; b Schar School of Policy and Government, George Mason University, Arlington, VA, USA; c Department of Medical Education, University of Toledo College of Medicine and Life Sciences, Toledo, OH, USA

**Keywords:** focused ultrasound, global surgery, health equity, histotripsy, leapfrog technology, surgical access

## Abstract

Five billion people, more than half of humanity, lack access to safe, affordable surgical care. While conventional surgical infrastructure requires decades and trillions of dollars to replicate in low-resource settings, emerging non-incisional technologies offer an alternative paradigm. Histotripsy is a focused ultrasound modality that mechanically destroys tissue without incisions, anesthesia, or prolonged hospitalization. It represents a potential leapfrog technology for addressing the global surgical divide. This editorial examines the technical, economic, and implementation considerations that position histotripsy as a transformative intervention for global surgical equity. We propose a framework for accelerated deployment and outline the policy, research, and financing mechanisms required to realize this potential within a generation.

## Introduction

### The surgical access crisis

The Lancet Commission on Global Surgery established that 5 billion people lack access to safe, affordable, and timely surgical care. This figure represents approximately 70% of the world’s population[1]. In many countries in Africa, the surgical workforce density stands at approximately 1 surgeon per 100 000 population, compared to 25 per 100 000 in high-income countries[2]. This disparity translates directly into preventable mortality. An estimated 18.6 million deaths annually are attributable to conditions requiring surgical intervention, with the vast majority occurring in low- and middle-income countries (LMICs)[3].

The traditional approach to addressing this gap is building conventional surgical infrastructure. This approach faces major barriers. A fully equipped operating theater costs $2–$5 million USD to construct and equip. It requires specialized power, water, and sterilization systems. It also depends on highly trained surgical teams, whose development requires 10–15 years of post-medical school training. Even with unlimited funding, replicating high-income surgical capacity in LMICs would take generations. Meanwhile, treatable conditions, including hepatocellular carcinoma, uterine fibroids, renal masses, and benign prostatic hyperplasia, continue to exact a devastating toll on populations that lack access to surgical intervention.

### Histotripsy: mechanism and clinical applications

Histotripsy represents a fundamentally different approach to tissue destruction compared to conventional heat-based methods. Unlike thermal ablation modalities that rely on heat deposition, histotripsy uses focused ultrasound to generate controlled acoustic cavitation at a precise focal point within tissue[4]. The resulting microbubble formation and collapse mechanically fractionates cells into acellular debris. The body’s immune system subsequently clears this debris[4]. The boundary between treated and untreated tissue is characteristically sharp, often sub-millimeter, because the cavitation threshold creates a binary response. Tissue either experiences complete destruction or remains entirely unaffected[5].

Clinical trials have demonstrated histotripsy’s efficacy across multiple organ systems. The THERESA trial established safety and effectiveness for hepatic tumor ablation. It reported 90-day complete response rates comparable to surgical resection in appropriately selected patients[6]. Investigations in renal, prostatic, and uterine pathology have similarly demonstrated promising outcomes[7]. Importantly, histotripsy procedures typically require only conscious sedation rather than general anesthesia. They eliminate the risk of blood loss inherent to incisional surgery, while also enabling same-day discharge in the majority of cases.

Regulatory status note: It is important to be explicit that, as of this writing, FDA clearance for histotripsy has been granted specifically for hepatic tumors. Investigations in renal, prostatic, and uterine pathology have demonstrated promising early results[7], but these applications remain investigational and have not received regulatory clearance in the United States or, to our knowledge, in any jurisdiction as of the submission of this editorial. Any LMIC deployment framework must account for this regulatory status carefully, as FDA clearance does not automatically confer approval under national regulatory agencies such as NAFDAC (Nigeria), the Ghana Food and Drug Authority, or equivalent bodies. Regulatory harmonization in LMICs may offer a pathway, but the timeline and scope remain uncertain.

### Reimagining the infrastructure requirements

The characteristics that make histotripsy attractive in high-resource settings include precision, minimal invasiveness, and rapid recovery. These characteristics become transformative when considered through the lens of global surgical access. Each barrier to conventional surgery in LMICs is directly addressed by histotripsy’s mechanism of action.

Surgical site infection rates in resource-limited settings range from 20 to 30%, compared to 2 to 5% in high-income countries. This reflects challenges with sterility, antibiotic availability, and post-operative care[8]. Histotripsy eliminates this risk category entirely. Intact skin provides a barrier against bacterial contamination. Moreover, reducing infections will decrease antibiotic use. This, in turn, will reduce the risk of antibiotic resistance. The global anesthesia workforce shortage, with fewer than 10 physician anesthesiologists in some LMICs serving populations of millions, becomes less constraining when procedures require only nurse-administered conscious sedation[9]. Blood banking infrastructure is expensive to maintain and carries disease transmission risks in some regions. Histotripsy obviates this need because the procedure causes no hemorrhage. Hospital bed constraints also ease when patients return home the same day rather than occupying limited inpatient capacity for postoperative recovery.

### The leapfrog paradigm

The mobile telecommunications revolution in Africa provides an instructive precedent. Rather than building expensive landline infrastructure, many African nations leapfrogged directly to mobile technology. They achieved 80% or higher penetration rates without the intermediate developmental step that characterized telecommunications evolution in wealthy countries[10]. Mobile money platforms subsequently enabled financial inclusion without brick-and-mortar banking infrastructure. This pattern suggests that developmental trajectories need not recapitulate historical sequences when disruptive technologies enable alternative pathways.

We propose that histotripsy represents an analogous opportunity for surgical care, with an important distinction from the telecommunications analogy: mobile phones were a mature, mass-market commodity technology at the time of their LMIC expansion. Histotripsy is an emerging technology in its early post-clearance phase. The leapfrog opportunity is real, but its realization requires deliberate investment in adaptation for resource-limited deployment rather than mere geographic replication of high-income implementations.

Cost projections (illustrative): A containerized histotripsy unit – comprising the treatment system, integrated ultrasound imaging, ruggedized power conditioning, and recovery space – could plausibly be deployed for approximately $400 000–$600 000 USD. This projection is hypothetical, with the listing of the Edison system at an estimated $300 000–$400 000; containerization, ruggedization, and power-stabilization systems at $50 000–$100 000; and initial consumable and maintenance reserves. These figures represent an illustrative order-of-magnitude projection requiring formal health-economic validation and presuppose manufacturer participation in tiered pricing models. Available modeling suggests that such units could perform 2500 or more procedures annually, at a cost of approximately $200–$400 per procedure, compared to $2000–$5000 for conventional surgery where available – bringing intervention within reach of microfinance, community insurance, and government subsidy programs[11].

### Training and implementation considerations

Histotripsy fundamentally alters the human capital requirements for procedural intervention. Conventional surgery requires a decade of postgraduate training to develop operative judgment and technical skill. Histotripsy operators require proficiency in ultrasound imaging, treatment planning, and procedure monitoring. The training timeline for these competencies has not yet been formally characterized for histotripsy specifically – this is an identified gap that the research community must prioritize. Based on analogous experience with ultrasound-guided and image-guided interventional procedures, training timelines are expected to be substantially shorter than those of open surgery^[^12,13^]^, but this assertion must be empirically validated through structured competency studies before large-scale LMIC deployment is planned. Virtual reality simulation platforms are increasingly validated for procedural training. They could enable standardized competency development with telemedicine supervision. This could connect operators at peripheral sites to expert oversight at regional centers of excellence[13].

This hub-and-spoke model mirrors successful implementation frameworks for other complex health interventions. The cascade training approach used to expand antiretroviral therapy access – training trainers who subsequently trained providers at increasingly peripheral levels – could be adapted to histotripsy deployment. The immediate feedback inherent to ultrasound-guided procedures accelerates competency development, as operators can visualize treatment effects in real time, which is likely to shorten the learning curve relative to conventional surgery. Prospective validation of these timelines must accompany any deployment initiative.

#### Sustainment and maintenance

A frank accounting of histotripsy deployment must address sustainment. Advanced medical devices deployed in LMICs have repeatedly failed, not because of initial inadequacy, but because of the absence of local maintenance capacity, reliable spare parts supply chains, and software support infrastructure – a pattern documented across imaging systems, dialysis equipment, and surgical devices. Containerized histotripsy units would require service-level agreements with manufacturers that include: on- or off-site training of biomedical engineering technicians; standardized consumable inventories maintained at regional depots; remote diagnostic and software update capabilities via telemedicine-enabled device monitoring; and clear escalation pathways for hardware failure. Regional maintenance hubs, analogous to the depot model used in successful vaccine cold-chain networks, could serve clusters of deployed units across catchment geographies. Procurement agencies, financing bodies, and health ministries should require manufacturers seeking LMIC contracts to demonstrate sustainment plans with a minimum 10-year service horizon as a condition of deployment. The field cannot afford another generation of expensive equipment that sits idle in district hospitals.

#### Policy and financing framework

Realizing histotripsy’s potential for global surgical equity requires coordinated action across multiple domains. First, device manufacturers must commit to equity-oriented design and tiered pricing structures. They should develop ruggedized, portable systems optimized for resource-limited deployment alongside premium products for high-income markets. Second, research funding agencies should prioritize implementation science. This includes clinical trials in LMIC settings, health economic analyses demonstrating cost-effectiveness, and studies of training models and maintenance protocols adapted to local conditions. Third, regulatory pathways require streamlining, potentially through regional harmonization initiatives that reduce duplicative approval processes while maintaining safety standards. Fourth, innovative financing mechanisms must be developed. A global fund for surgical innovation, analogous to the Global Fund for AIDS, Tuberculosis, and Malaria, could mobilize the $2–$3 billion over a decade required to deploy histotripsy infrastructure at scale[11]. Blended finance models, public-private partnerships, philanthropic capital, and impact investing offer realistic and sustainable pathways^[^14,15^]^. Finally, sustained advocacy is essential. Surgical access must achieve parity with infectious disease and maternal health on the global health agenda.

## Conclusion

The five billion people currently lacking surgical access deserve more than incremental progress. They deserve the same transformative interventions that redefined survival in wealthy nations. Histotripsy will not solve every surgical need. Trauma, obstetric emergencies, and complex reconstructive procedures will continue to require conventional operating theaters. However, for the substantial burden of disease amenable to tissue ablation, including liver tumors, fibroids, and prostatic obstruction, histotripsy offers a realistic pathway to equity within a generation, provided that the technology matures through its investigational pipeline, sustainment infrastructure is built in parallel with device deployment, and manufacturers and financiers commit to genuine equity-oriented models.

The technology exists and is advancing. The need is desperate and documented. The remaining question is whether we possess the collective will to bridge them, and whether the global health community will insist that enthusiasm for innovation be matched by rigor in implementation.

## Data Availability

This editorial did not involve the collection or analysis of primary data. All data referenced are from publicly available, peer-reviewed sources cited in the manuscript.
